# Single-Cell Transcriptomic Profiling of MAIT Cells in Patients With COVID-19

**DOI:** 10.3389/fimmu.2021.700152

**Published:** 2021-07-30

**Authors:** Jijing Shi, Jianglin Zhou, Xiaochang Zhang, Wei Hu, Jin-Fang Zhao, Shengqi Wang, Fu-Sheng Wang, Ji-Yuan Zhang

**Affiliations:** ^1^Key Medical Laboratory of Stem Cell Transformation and Application, The First People’s Hospital of Zhengzhou, Zhengzhou, China; ^2^Department of Infectious Diseases, Fifth Medical Center of Chinese People’s Liberation Army (PLA) General Hospital, National Clinical Research Center for Infectious Diseases, Beijing, China; ^3^Beijing Institute of Radiation Medicine, Beijing, China; ^4^Medical School of Chinese People’s Liberation Army (PLA), Beijing, China

**Keywords:** COVID-19, MAIT, single-cell RNA sequencing, disease severity, pyroptosis

## Abstract

**Background:**

Mucosal-associated invariant T (MAIT) cells are considered to participate of the host immune response against acute severe acute respiratory syndrome coronavirus 2 (SARS-CoV-2) infection; however, single-cell transcriptomic profiling of MAIT cells in patients with COVID-19 remains unexplored.

**Methods:**

We performed single-cell RNA sequencing analyses on peripheral MAIT cells from 13 patients with COVID-19 and 5 healthy donors. The transcriptional profiles of MAIT cells, together with assembled T-cell receptor sequences, were analyzed. Flow cytometry analysis was also performed to investigate the properties of MAIT cells.

**Results:**

We identified that differentially expressed genes (DEGs) of MAIT cells were involved in myeloid leukocyte activation and lymphocyte activation in patients with COVID-19. In addition, in MAIT cells from severe cases, more DEGs were enriched in adaptive cellular and humoral immune responses compared with those in moderate cases. Further analysis indicated that the increase of cell cytotoxicity (killing), chemotaxis, and apoptosis levels in MAIT cells were consistent with disease severity and displayed the highest levels in patients with severe disease. Interestingly, flow cytometry analysis showed that the frequencies of pyroptotic MAIT cells, but not the frequencies of apoptotic MAIT cells, were increased significantly in patients with COVID-19, suggesting pyroptosis is one of leading causes of MAIT cell deaths during SARS-CoV-2 infection. Importantly, there were more clonal expansions of MAIT cells in severe cases than in moderate cases.

**Conclusions:**

The results of the present study suggest that MAIT cells are likely to be involved in the host immune response against SARS-CoV-2 infection. Simultaneously, the transcriptomic data from MAIT cells provides a deeper understanding of the immune pathogenesis of the disease.

## Introduction

Acute infection with severe acute respiratory syndrome coronavirus 2 (SARS-CoV-2) has rapidly caused the ongoing worldwide pandemic of coronavirus disease 2019 (COVID-19). COVID-19 can present with a spectrum of illness, from asymptomatic, mild, moderate, to severe and death (1, 2). SARS, Middle East respiratory syndrome (MERS), and COVID-19 are the three epidemics of lethal diseases caused by coronaviruses during the last twenty years. SARS-CoV and MERS−CoV are highly pathogenic and can cause severe diseases presented as acute respiratory distress syndrome. SARS-CoV-2 seems to be less virulent but shows more infectious transmission from human to human through air droplets from the respiratory tract compared with that in SARS-CoV or MERS-CoV, with mortality rates of 3.4%, 9.6%, and ~35%, respectively (3, 4).

The outcome of acute viral infection is mainly influenced by three factors: Viral, immune factors, and viral-host interactions. Acute SARS-CoV-2 infection and the antiviral host immune responses influence and interact with each other *in vivo*, shaping different disease severities and outcomes. Lymphopenia and cytokine release syndrome, as well as dyspnea, hypoxemia, and acute respiratory distress, are often found in patients with severe COVID-19, suggesting that the host immune responses against the viral infection play an important role in the development of pneumonia in COVID-19 cases (5). Therefore, it is of the utmost importance to understand the immunopathology of the disease, which would also help to identify effective drugs or develop efficient prophylactic vaccines for the disease. Recent studies have shown that severe inflammatory responses, impaired innate and adaptive immune responses were characterized in patients with severe COVID-19 (6–12). Furthermore, based on single−cell RNA sequencing (scRNA-seq), the transcriptomic properties of adaptive immunocytes, such as T cells and B cells (13, 14), as well as classical innate immune cells, including monocytes, natural killer cells, and neutrophils, have been identified. These studies deepen our understanding of the disease’s immunopathology. However, scRNA profiling of innate-like T cells, such as mucosal-associated invariant T (MAIT) cells, remains unexplored in patients with COVID-19.

MAIT cells are recently defined innate-like T cells that are mainly distributed in the blood, intestinal laminae lymphatic tissues, liver, and lungs (15, 16). MAIT cells express a semi-invariant T cell receptor (TCR) mostly containing the relatively conserved Vα7.2-Jα33 chain, preferentially paired with a restricted Vβ2 or Vβ13 repertoire, and a C-type lectin-like receptor, CD161 (17–20). MAIT cells can be considered to span both the innate and adaptive arms, and play an important role in the innate host defense against various bacterial and viral infections (e.g., influenza virus, hepatitis B virus, hepatitis C virus, hepatitis D virus, as well as human immunodeficiency virus type 1), through secreting effector molecules, including interferon gamma (IFN-γ), tumor necrosis factor alpha (TNF-α), Granzyme B, perforin, and interleukin 17 (IL-17) (17, 21–24). More recently, Parrot et al. and Jouan et al. observed certain phenotypical and functional alterations of MAIT cells in patients with COVID-19 that were associated with disease severity (25, 26). In the present study, we performed scRNA-seq analyses to characterize MAIT cells in peripheral blood mononuclear cells (PBMCs) from patients with varying severities of COVID-19, and depicted an unbiased and comprehensive visualization of blood MAIT cells in the progression of the disease, which will lead to a better understanding of the pathogenic mechanism of COVID-19.

## Materials and Methods

### Data Collection and MAIT Extraction

The raw scRNA-seq FASTQ files of PBMCs from 13 patients and 5 healthy controls were downloaded from the Genome Sequence Archive of the Beijing Institute of Genomics (BIG) Data Center (http://bigd.big.ac.cn/gsa-human, accession HRA000150). The 13 patients with COVID-19 were classified into three clinical conditions: moderate (n = 7), severe (n = 4) and convalescent (conv; n = 6, of whom 4 were paired with moderate cases). The downloaded reads were then processed individually using the Cell Ranger (v.4.0.0, 10xgenomics, https://www.10xgenomics.com/) count pipeline with the GRCh38 human reference genome to generate gene expression matrices. The subsequent analyses were performed by R (v.4.0.2) scripts with the Seurat (v.3.2.2) package as described in our previous study (13). Briefly, the Cell Ranger output filtered gene expression matrices were further filtered according to the parameters in our previous study (13). The genes expressed at a percentage of 0.1% cells or more were kept for every sample. The cells were filtered as following criteria: (1) the number of genes is equal to or larger than 500; (2) the number of unique molecular identifiers (UMIs) is equal to or larger than 800; (3) the percentage of the mitochondrial genes is no more than 10%. Those cells that did not satisfy the above criteria were removed. Then, the datasets from different samples from four conditions were integrated into an integrated and unbatched dataset using the “standard workflow”, as described at https://satijalab.org/seurat/v3.2/integration.html. The integrated dataset was scaled and principal components analysis (PCA) was calculated. The top 20 principal components (PCs) were selected to construct a shared nearest neighbor (SNN) network and an unsupervised graph-based clustering approach, the Louvain algorithm, was applied to cluster cells with a parameter resolution = 1.5. Clusters were then classified and annotated based on the selected classic markers (13). Specifically, MAIT cells were located using marker *SLC4A10* (encoding solute carrier family 4 member 10) and *TRAV1-2* (encoding T cell receptor alpha variable 1-2) and were extracted to another dataset to perform downstream analyses. Finally, uniform manifold approximation and projection (UMAP) was applied to visualize the clustering result in a two-dimensional space (27).

### Identification of Differentially Expressed Genes (DEGs) and GO Enrichment

To identify the DEGs across different clusters and/or conditions, the “FindMarker” function in the Seurat package was performed with multiple threshold parameters, including an average log2 (fold change) ≥ 0.5, a Benjamini–Hochberg-corrected *P* value ≤ 0.01, and detection in ≥ 10% of cells in at least one condition. The obtained DEGs were uploaded to the Metascape webtool (www.metascape.org) (28) and the gene sets derived from gene ontology (GO) Biological Process Ontology were selected to obtain their function profiles.

### Defining Cell State Scores

We used cell scores to evaluate the degree to which individual cells express a certain pre-defined gene set (13, 29, 30). For a given gene set (*G_j_*) reflecting a specific cell state or biological function, the score for every cell *i*, *SC_j_(i)*, quantifying the relative expression of *G_j_* in cell *i* as the average relative expression (*Er*) of genes in *G_j_* compared to the average relative expression of a control gene set (*G_j_* ^cont^): *SC_j_(i)* = average[*Er*(*G_j_, i*)] - average[*Er*(*G_j_* ^cont^ *, i*)]. The control gene set was defined by first binning all the analyzed genes into 25 bins of aggregate expression levels. For each gene in the given gene set, we randomly chose 100 genes from the same expression bin. The “AddModuleScore” function in Seurat package was used to implement this approach with “nbin” set to 25. We respectively used CELL KILLING (GO:0001906), CELL CHEMOTAXIS (GO:0060326), APOPTOTIC SIGNALING PATHWAY (GO:0097190), 9 activation-related genes (*CD38, CD69, CD25(IL2RA), CD95(FAS), CD134(TNFRSF4), CD137(TNFRSF9), CD154(CD40LG), MKI67, KLRG1*) to define the cell killing, cell chemotaxis, apoptosis, and the activation score.

### TCR Analysis

The Cell Ranger (v.4.0.0) vdj pipeline with GRCh38 as the reference was used to perform the gene quantification and TCR clonotype assignment. Then, the files with contig annotations and clonotype frequencies were obtained. According to our previous study, only cells with at least one productive TCR α-chain (TRA) and one productive TCR β-chain (TRB) were kept for further analysis (13). The cell barcodes and sample identifiers were concatenated to define the cell identifiers and to associate the gene expression data with TCR information for each MAIT cell. Here, a clonotype refers a unique TRA(s)-TRB(s) pair (complementary determine region 3 (CDR3) amino acid sequences included). If the cell numbers of one clonotype were greater than 1, this clonotype was considered to be clonal, and the number indicated the degree of clonality of the clonotype.

### Flow Cytometry

MAIT cells were identified as CD161^hi^TCR Vα7.2^+^ cells among CD3^+^ T cells. To detect surface markers, the following antibodies were used: anti-CD3-APC-Cy7 (BD Biosciences, San Diego, California, USA. Clone: OKT3), anti-CD161-PE (BD Biosciences. Clone: HP-3G10), and anti-TCR Vα7.2-BV421 (BD Biosciences. Clone: 3C10). For fluorochrome-labeled inhibitors of caspases (FLICA) caspase-1 detection, FLICA staining was conducted in accordance with the manufacturer’s instructions (Bio-Rad, Hercules, CA, USA). Cells were incubated with the FLICA caspase-1 reagent for 1 hour at 37 C and then cells were washed for downstream surface markers staining and intracellular anti-capase-3-Alexa 647 (BD Biosciences. Clone: C92-605). Data were acquired on a BD FACSCanto II flow cytometer (BD Biosciences), and further analyzed using FlowJo (Ashland, OR, USA) Tree Star software.

### Boxplot and Comparisons

All the boxplots in this study were plotted using “geom_boxplot” in ggplot2 (ggplot2: Elegant Graphics for Data Analysis. Springer-Verlag New York, 2016) R package. Every dot represented a sample. The horizontal line within each box acted as the median, and the bottom and top of each box indicated the 25-th and 75-th percentile. The Wilcoxon rank-sum test was applied to test the significance of the difference between conditions using the “geom_signif” function in ggsignif (Significance Brackets for ‘ggplot2’) R package. The statistical tools and methods for each analysis are explicitly described in the figure legends.

## Results

### Single-Cell Transcriptional Profiling of Peripheral MAIT Cells

**Figure 1:**
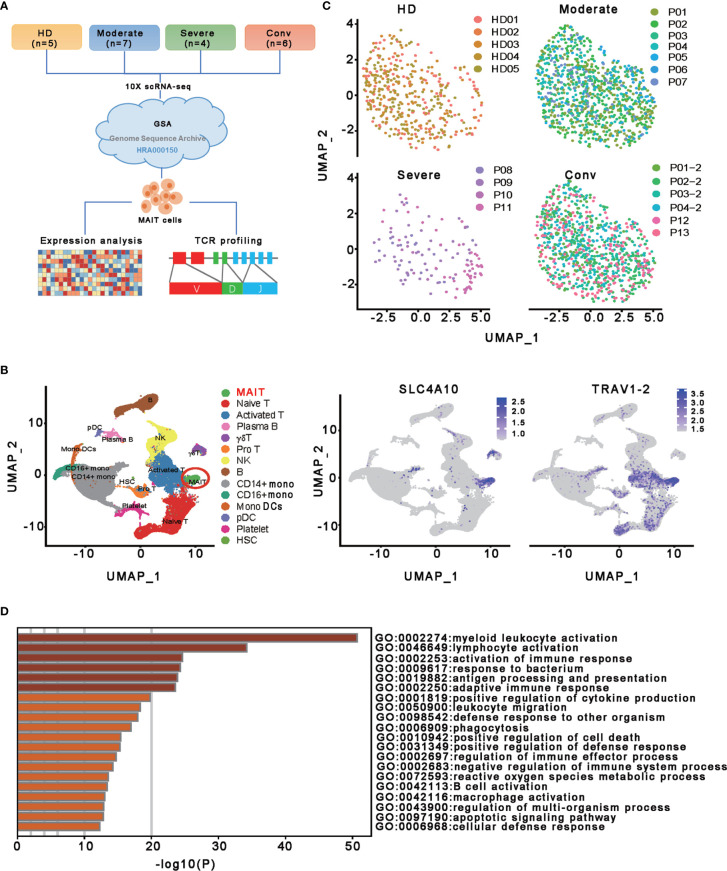
Single-cell gene expression profiling of MAIT cells derived from PBMCs of the participants. **(A)** Schematic diagram of this study design. The scRNA−seq data for MAIT cells were processed for expression analysis and TCR profiling across four conditions, including HD (n = 5), moderate (n = 7), severe (n = 4) and conv (n = 6). **(B)** UMAP projection of integrated single-cell transcriptomes of 121464 cells from all participants (left) and MAIT cell annotation (right). The left scatter plot shows all 14 cell types and each dot represents a single cell, colored according to cell type. MAIT cells are circled in red. The right two scatter plots show the expression of canonical markers of MAIT cells and the cell are colored according to the expression level. **(C)** UMAP representations of MAIT cells in HD, moderate, severe, and conv conditions, respectively. Cells are color-coded by individual samples, respectively. **(D)** Gene enrichment analyses of the DEGs in the MAIT cells in comparison with 13 other immune cell types (naïve T cell, activated T cells, γδ T cells, proliferative T cells, natural killer cells, B cells, plasma B cells, CD14+ monocytes, CD16+ monocytes, monocyte-derived dendritic cells, plasmacytoid dendritic cells, platelets, and hemopoietic stem cells) in PBMCs.

To characterize the transcriptional profiling of MAIT cells in patients with COVID-19, we analyzed the published scRNA-seq datasets of PBMCs from thirteen patients and five healthy donors (HDs) (13) (**Figures 1A, C** **)**. The 13 patients with COVID−19 consisted of moderate cases (n = 7), severe cases (n = 4), and convalescent (n=6; of whom 4 were paired with the moderate cases) (**Figure 1C**). Fourteen cell types comprising a total of 121464 cells were selected and annotated after quality control and doublets removal. Specifically, according to the expression level of canonical markers *SLC4A10* and *TRAV1-2*, a total of 2502 MAIT cells were identified and extracted for subsequent detailed analyses (**Figures 1B** and **S1A**). Among these MAIT cells, 417 cells (16.7%) were from HDs, 1019 cells (40.7%) were from moderate cases, 130 cells (5.2%) were from severe cases, and 936 cells (37.4%) were from convalescent samples. Meanwhile, there were an average of 1.97%, 2.58%, 0.53%, and 2.53% MAIT cells among PBMCs per sample in the HD, moderate, severe, and conv groups, suggesting profound depletion of MAIT cells in patients with severe disease. The changed MAIT proportions in the different conditions agreed with the previous findings (13, 25, 26). Visualization of MAIT cells *via* UMAP clearly demonstrated their batch-less nature and comparability (**Figure 1C**). As such, we clearly defined the MAIT cells in the peripheral blood of individuals with COVID-19.

**Figure 2:**
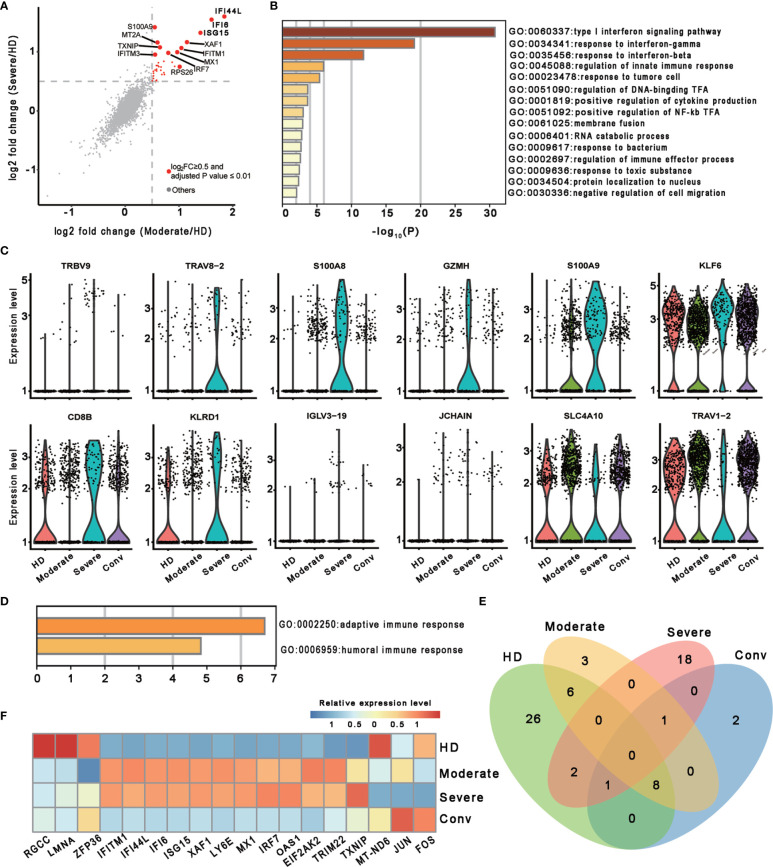
Characterization of MAIT cells in individuals across four conditions. **(A)** Scatter plot showing the comparisons of DEGs in the MAIT cells between severe (or moderate) and HD controls. The red dots represent significantly upregulated genes with an adjusted *P* value ≤ 0.01 and average log_2_ fold change ≥ 0.5. Example genes are labeled with gene symbols. The gray dots represent genes with adjusted *P* > 0.01 or average log2 fold change < 0.5. A two sided unpaired Mann–Whitney U-test was applied. *P* values were adjusted using Bonferroni correction. **(B)** Gene enrichment analyses of the significantly upregulated DEGs in **(A)**. GO terms are labeled by name and ID, and sorted by -log10(P) values. A smaller P value was expressed in a darker color. **(C)** Violin plots showing gene expression levels of DEGs of MAIT cells in the severe group (n = 4) in comparison with their counterparts in the moderate group (n = 7). **(D)** Gene enrichment analyses of the DEGs in **(C)**. Display settings are similar to **(B)**. **(E)** Venn diagram showing the overlapping DEGs among the HD (n = 5), moderate (n = 7), severe (n = 4) and conv (n = 6) conditions. **(F)** Heatmap showing the relative expression level of shared genes of **(E)** in at least two conditions. Rows denotes the four conditions and columns denote the shared genes.

To investigate the transcriptomic features of MAIT cells comprehensively, we first compared the expression patterns of MAIT cells with 13 other immune cell types, including naïve T cell, activated T cells, γδ T cells, proliferative T cells, natural killer cells, B cells, plasma B cells, CD14+ monocytes, CD16+ monocytes, monocyte−derived dendritic cells, plasmacytoid dendritic cells, platelets, and hemopoietic stem cells, in PBMCs and identified a total of 230 differentially expressed genes (DEGs). Further analysis found that these DEGs were mainly involved in myeloid leukocyte activation, lymphocyte activation and migration, antigen processing and presentation, and cytokine production (**Figure 1D**), suggesting that MAIT cells may engage in the immune response against SARS-CoV-2. Second, we compared the expression patterns of MAIT cells from patients with moderate or severe disease with the counterparts from HDs. We found that DEGs were mostly enriched in the type I interferon signaling pathway, response to interferon (gamma and/or beta), regulation of innate immune response, positive regulation of cytokine production, and NF-kappaB transcription factor activity (TFA) processes (**Figures 2A, B**). These results agreed with the previously observed results in 13 other immune cell types from patients with COVID-19 (13, 31). Third, we compared the transcriptional profiles of MAIT cells from patients with severe disease with the counterparts from patients with moderate disease to explore the correlation of disease severity and gene expression in MAIT cells. A total of twelve DEGs were recognized. Ten of them (*TRBV9*, *TRAV8-2*, *S100A8*, *GZMH*, *S100A9*, *KLF6*, *CD8B*, *KLRD1*, *IGLV3-19*, and *JCHAIN*) were upregulated, whereas the only two were downregulated genes (*SLC4A10* and *TRAV1-2*), which are canonical markers of MAIT cells (**Figure 2C**). Further analysis found that the DEGs were mainly enriched in adaptive immune response and humoral immune response (**Figure 2D**), suggesting that the expression of these DEGs might correlate with the disease severity. Interestingly, we found that 18 DEGs were in at least two conditions (**Figure 2E**). Specifically, twelve of them (*IFITM1*, *IFI44L*, *IFI6*, *ISG15*, *XAF1*, *LY6E*, *MX1*, *IRF7*, *OAS1*, *EIF2AK2*, *TRIM22*, and *TXNIP*) showed increased expression during COVID−19 and later declined in the convalescent condition; however, six DEGs (*RGCC*, *LMNA*, *ZFP36*, *MT-ND6*, *JUN*, and *FOS*) showed the opposite; i.e., the expression levels of these genes were reduced during infection with COVID-19 and recovered after being restored to health (**Figure 2F** and **Table S1**). These findings suggested that the encoded proteins might be involved in the virus-host interaction and host immune response, thus reflecting disease severity.

### Features of MAIT Cells in Patients With COVID-19

**Figure 3:**
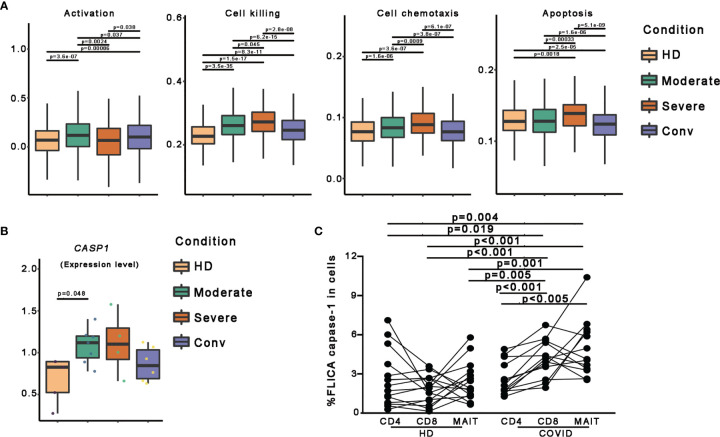
Pyroptotic MAIT cells increased in patients with COVID-19. **(A)** Box plots of the expression levels of genes associated with four GO biological process in MAIT cells derived from the HD (n = 5), moderate (n = 7), severe (n = 4), and conv (n = 6) samples. Conditions are displayed in different colors. Horizontal lines represent median values, with whiskers extending to the farthest data point within a maximum of 1.5 × the interquartile range. **(B)** The expression level of the *CASP1* gene in participants from four conditions. A dot represents a single sample. Samples and their corresponding conditions are displayed in different colors. **(C)** Comparisons of FLICA caspase-1 in peripheral CD4^+^ T, CD8^+^ T and MAIT cells from healthy donors and patients with COVID-19. Statistical analysis was performed using SPSS software version 22 (IBMCorp., Armonk, New York, USA). For comparison, the Mann–Whitney U test was used for comparisons between healthy donors and patients with COVID-19. A paired Student’s t-test was adopted for the analysis of CD4^+^ T, CD8^+^ T and MAIT cells in the same group. p values < 0.05 indicated a significant difference. HD, healthy donor.

To further investigate the features of MAIT cells in patients with COVID-19, we used a scoring system to evaluate certain key biological processes, such as activation, cell killing, cell chemotaxis, and apoptosis. Optimal cell activation is a prerequisite for cell function, and we found that MAIT cells in patients with COVID-19 showed trends if higher activation levels than in HDs, suggesting a consistent response by MAIT cells to SARS-CoV-2 infection. Notably, the activation levels of MAIT cells in severe cases were less than those in moderate and conv cases. For functional evaluation, we found that the cell killing level in MAIT cells increased consistently with disease severity and displayed the highest levels in severe cases. Importantly, cell chemotaxis and apoptosis levels in MAIT cells were also highest in severe cases, suggesting that the increased migration and cell death pathways in the MAIT cells of patients with severe COVID-19 may be associated with their MAIT-cell loss in peripheral blood (**Figure 3A**).

To investigate the association between pyroptosis, a newly identified programmed cell death pathway, and disease severity during SARS-CoV-2 infection, we first compared *CASP1* gene expression and found the *CASP1* gene expression in patients with COVID-19 showed higher levels than those in in HDs (**Figures 3B** and **S2**). To confirm this finding, we used FLICA to detect the percentages of the active form of caspase-1^+^ cells. We observed that the frequencies of FLICA caspase-1^+^ MAIT cells were significantly increased in the peripheral blood of patients with COVID-19. By contrast, the frequencies of active caspase-3^+^ MAIT cells was not significantly increased in patients with COVID-19 (**Figures 3C** and **S3**). Interestingly, CD8 T cells, but not CD4 T cells, also showed an increased pyroptotic phenotype in patients with COVID-19 (**Figures 3C** and **S3**). Collectively, these data, at least in part, confirm that MAIT cell pyroptosis occurs in SARS-CoV-2 infection and pyroptosis as possible cause of MAIT cell loss.

### Clonal Expansion of MAIT Cells and Preferred Usage of V(D)J Genes in Patients With COVID-19

**Figure 4:**
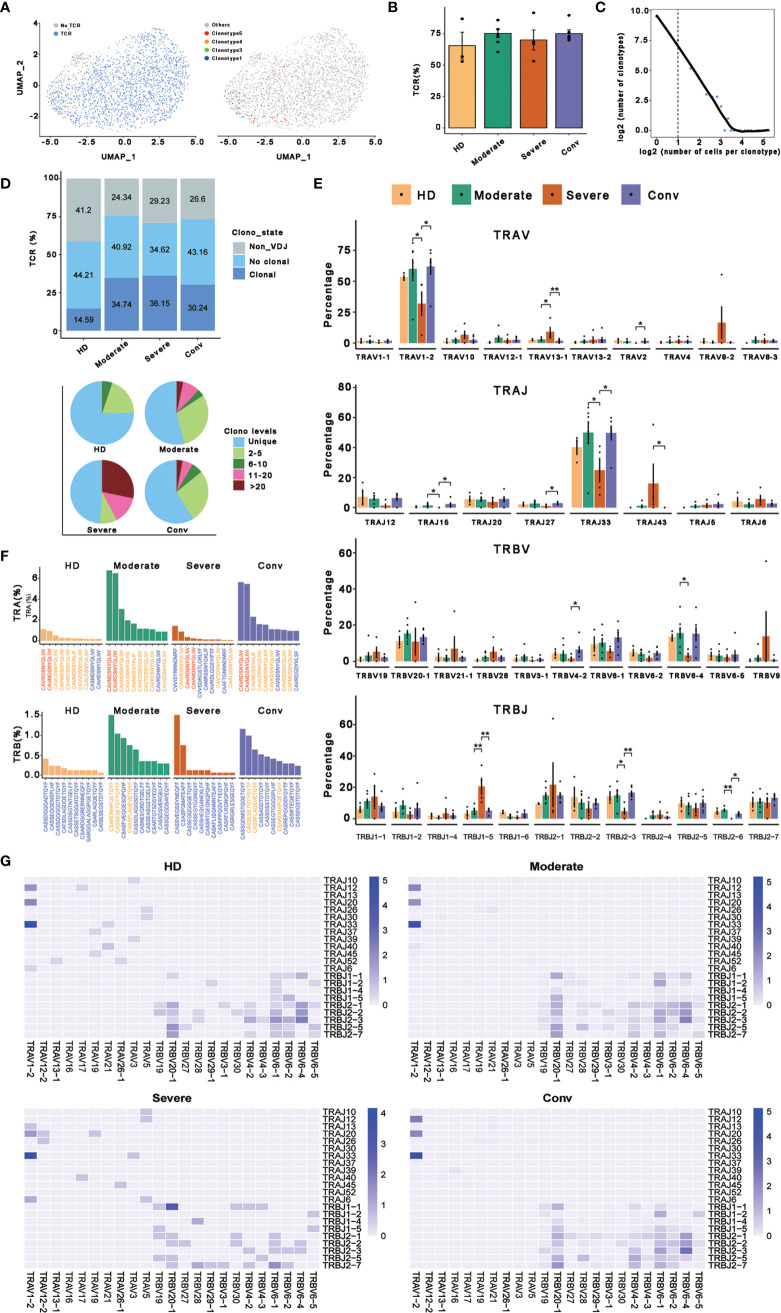
Expanded TCR clone and selective V(D)J gene usage in MAIT cells. **(A)** UMAP of MAIT cells derived from PBMCs. Cells detected with TCRs are highlighted in blue (left) and the distributions of some types of TCR are shown (right). **(B)** Bar plots showing the percentage of TCR detection in each MAIT cell condition. **(C)** The association between the number of MAIT cell clones and the number of cells per clonotype. Nonclonal cells are on the left of the dashed line and clonal cells are on the right. These points were fitted by LOESS, which shows the negative correlation between the two axes. **(D)** The percentages of the clone states of MAIT cells in each condition and the distribution of different levels of clonal MAIT cells across four conditions. **(E)** The percentages of usage of some *TRAV*, *TRAJ*, *TRBV*, and *TRBJ* genes across four conditions in MAIT cells, in which error bars are represented by ± s.e.m. Differences in all comparisons were analyzed by the Mann-Whitney test and the results of *P* < 0.05 are labeled over the bar plots (0.01 < *P* < 0.05 is marked by ‘*’ and 0.001 < *P* < 0.01 is marked by ‘**’). **(F)** The percentages of top ten CDR3 usages in MAIT cells across four conditions are shown. Sequences in blue are distinctive usages compared with other conditions. Sequences in red are common usages across the four conditions. Sequences in orange are common usages in at least two conditions. **(G)** TRA/B rearrangement differences in each MAIT cell condition. The color legends in the right of these plots indicate the usage percentage of specific V-J gene pairs.

To better determine the clonal relationship among individual MAIT cells and their usage of V(D)J genes across the four conditions, we reconstructed TCR sequences of MAIT cells from the TCR sequencing of 20 cases. In this analysis, 1687 cells matching TCR information were detected from 2318 MAIT cells and the percentage of cells with TCR information is more than 70% in all conditions, except for the HD (**Figures 4A, B**). We then performed statistical processing of all clonotypes (**Figures 4C, D**). Overall, the number of clonotypes was negatively logarithmically correlated with the number of cells per clonotype (**Figure 4C**). It should be noted that the number of high frequencies of clonotypes of MAIT cells was relatively high. Compared with the HDs, clonal expansion was obvious in patients with COVID-19 both in disease progression and in convalescence (**Figure 4D**). Interestingly, there were more large clonal expansions (clonal size >10) in the severe cases than in the other conditions, indicating that some clonotypes might be abnormally over expanded in the MAIT cells of severe cases. To study the abnormality and gene preference of TCRs in MAIT cells of severe cases, we compared the usage of V(D)J genes across the four conditions (**Figures 4E–G**). The top 10 complementarity determining region 3 (CDR3) sequences in TRB were quite different across the four conditions (**Figure 4E**) and the moderate and conv conditions shared three CDR3 sequences because four samples from these conditions were paired. However, for all the top 10 CDR3 sequences across the four conditions, there were only 21 unique CDR3 sequences in TRA. In total, the usage percentage of the top 10 CDR3 sequences in the HD condition was lower and more balanced compared with those of the other three conditions. Notably, we found that the percentage of cells using *TRAV1−2* or *TRBV33* was much higher than that of other genes in TRA and the percentage in severe cases was significantly decreased compared with that in the moderate and conv cases (**Figure 4E**). *TRAV1-2* is a marker gene of MAIT cells and it was also expressed at a lower level inside MAIT cells of severe cases compared with that in the other conditions. We also found that the usage levels of the gene pairs *TRAV1-2* and *TRAJ33*, and *TRAV1-2* and *TRAJ20* were high across all conditions (**Figure 4G**). Besides, the usage level of gene pair *TRAV1-2* and *TRAJ12* was also high across all conditions except for that in the severe cases. Thus, it seems that in TRA, these three gene pairs were mainly used only in moderate and conv cases. The lost gene pair *TRAV1-2* and *TRAJ12* might influence MAIT cell antigen recognition of patients with severe disease.

## Discussion

Previous studies have revealed that MAIT cell numbers decreased with disease severity and were later restored in the peripheral blood of convalescent patients (13), but were highly enriched in the airways of patients with severe disease, coupled with strong activation (25). Importantly, higher numbers of CD69^+^ peripheral blood MAIT cells in patients with COVID-19 on admission was predictive of the clinical course and disease severity (26). These findings hinted that MAIT cells are involved in the host immune response against SARS-CoV-2 and are possibly engaged in COVID-19 immune damage, even pulmonary fibrosis (32). As such, investigating the precise transcriptomic profiling of MAIT cells, as well as their relationships with disease severity in patients with COVID-19, is of great importance to understanding COVID-19 progression and to develop effective therapy (33). To address this issue, in the present study, we profiled the transcriptome of MAIT cells in patients with COVID-19 at single-cell resolution.

Our study provided an unbiased visualization of MAIT cells in the peripheral blood of patients with COVID-19. We found that DEGs in MAIT cells were mainly involved in myeloid leukocyte activation, lymphocyte activation, and antigen processing and presentation, suggesting their unique response to SARS-CoV-2 infection. Furthermore, an intensive interferon response was observed in patients with COVID-19 in MAIT cells, exemplified by the high enrichment of biological processes related to DEGs under disease conditions. Moreover, additional DEGs in MAIT cells from patients with severe disease were enriched in adaptive and humoral immune responses. With regard to functional evaluation, the cell killing level in MAIT cells increased consistently with increasing disease severity and displayed the highest levels in patients with severe disease, suggesting that the enhanced cytotoxicity of MAIT cells might be associated with immune-induced damage, particularly in patients with severe COVID-19. Notably, we observed that the activation levels of MAIT cells in severe cases were lower than those in moderate and convalescent cases, which conflicts with the results other studies that identified MAIT cells with high expression of the CD69 activation marker being associated with poor clinical outcome and disease severity in patients with COVID-19 (25, 26). The different activation marker profiles of MAIT cells could be explained by the fact that the activation of MAIT cells was analyzed at the protein level in previous studies, which is different from RNA level scoring system in our study. Therefore, whether the discrepancy is associated with various regulations during or after transcription, or whether the discrepancy could predict the clinical outcomes of patients with COVID−19 requires further investigation Finally, MAIT cells experienced distinctive TCR clonal expansion, evidenced by increased MAIT cells clonality in non-healthy condition and a biased usage of *TRAV* and *TRAJ* genes. Overall, MAIT cells manifested a complex and specific transcriptional profile in patients with COVID-19, suggesting their possible engagement in the immune response against SARS-CoV-2 infection.

We and other groups found that the frequencies of peripheral MAIT cells were persistently decreased in patients with severe COVID-19, which might have detrimental consequences for the immune defense against microbial disease and immune homeostasis at barrier sites (25). However, the factors that led to the obvious decrease of MAIT cells in peripheral blood of patients with COVID-19, especially in severe cases, are not clear. Our data present the transcriptomic profiling of peripheral MAIT cells in patients with COVID-19, which might provide hints to the cause of the loss of MAIT cells. First, the cell chemotaxis level in peripheral MAIT cells was observed to be the highest in patients with severe disease, suggesting the increased migration of MAIT cells, which might be consistent with other studies revealing that the enrichment of MAIT cells in airways may lead to an marked decrease of MAIT cells in the peripheral blood of patients with COVID-19 (25, 26, 33). Second, the increased cell death of MAIT cells because of enhanced apoptosis might also contribute to the decline of peripheral MAIT cells in patients with severe COVID-19. Third, pyroptosis is a recently defined programmed cell death characterized by active caspase-1-mediated gasdermin−D (GSDMD) cleavage and subsequent plasma membrane instability, leading to emission of proinflammatory signals, including signature cytokines IL-1β and IL-18, which activate intense inflammation (34–36). We found that the frequencies of pyroptotic MAIT cells, but not the frequencies of apoptotic MAIT cells, were significantly increased in the peripheral blood of patients with COVID-19. These data, at least in part, confirm that pyroptosis is one of leading causes of deaths of MAIT cells in SARS-CoV-2 infection. In the future, we need to know how to maintain the homeostatic MAIT cells in patients with COVID-19. Moreover, it is virtually important to identify MAIT cells as our friend or foe in patients with COVID-19, and to explore new methods to modulate MAIT cells to keep the balance of advantages and disadvantages of MAIT cells. More recently, our studies have demonstrated that intravenous umbilical cord-derived mesenchymal stem cell infusion in patients with moderate and severe COVID-19 is safe and well tolerated (37, 38). Of note, further studies should investigate the factors underling T-cell pyroptosis, such as MAIT cells and CD8 T cells, in patients with COVID-19.

In summary, the transcriptomic profiling of MAIT cells depicted in this study provided substantial value to further clarify the involvement of MAIT cells in COVID-19 immunopathogenesis, and might be helpful to evaluate their potential as biomarkers and/or immune intervention targets.

## Data Availability Statement

The datasets presented in this study can be found in online repositories. The names of the repository/repositories and accession numbers can be found below, http://bigd.big.ac.cn/gsa-human, HRA000150.

## Ethics Statement

The studies involving human participants were reviewed and approved by the Ethics Committee of the Fifth Medical Center of PLA General Hospital. The patients/participants provided their written informed consent to participate in this study.

## Author Contributions

J-YZ, F-SW, SW, and JS conceived and designed the study. JZ and XZ analyzed the data. WH performed the experiments. JS, JZ, XZ, WH, J-FZ, SW, F-SW, and J-YZ wrote the article. All authors contributed to the article and approved the submitted version.

## Funding

This work was supported by Young and middle-aged health science and technology innovation talent project of Henan Province (YXKC2020060), the Innovative Research Team in the National Natural Science Foundation of China (81721002), and grants from the National Natural Science Foundation of China (No. 81830101).

## Conflict of Interest

The authors declare that the research was conducted in the absence of any commercial or financial relationships that could be construed as a potential conflict of interest.

## Publisher’s Note

All claims expressed in this article are solely those of the authors and do not necessarily represent those of their affiliated organizations, or those of the publisher, the editors and the reviewers. Any product that may be evaluated in this article, or claim that may be made by its manufacturer, is not guaranteed or endorsed by the publisher.

## Supplementary Material

Supplementary Figure 1 Quality of clustering and annotation of MAIT cells. **(A)** MAIT cell annotation and UMAP projection of integrated single-cell transcriptomes of 121464 cells from all participants. The left two violin plots show the expression distribution of MAIT cell canonical markers in all clusters. The right scatter plot shows all 14 cell types and each dot represents a single cell, colored according to cell type. MAIT cells are circled in red. **(B)** UMAP projections of gene counts (left), UMI counts (middle), and the percentage of mitochondrial genes (right) in MAIT cells. **(C)** Violin plots depicting the distribution of gene counts (left), UMI counts (middle), and the percentage of mitochondrial genes (right) in MAIT cells.

Supplementary Figure 2 Dynamic transcriptional profile of MAIT cells. Heatmap showing the relative expression levels of selected markers in different samples. Rows represent shared genes and columns represent samples from different conditions.

Supplementary Figure 3 Comparisons of active capase-3 in peripheral CD4^+^ T, CD8^+^ T and MAIT cells from healthy donors and COVID-19 patients. Statistical analysis was performed using SPSS software version 22 (IBMCorp., Armonk, New York, USA). For comparison, the Mann–Whitney U test was used for comparisons between healthy donors and patients with COVID-19. A paired Student’s t-test was adopted for the analysis of CD4^+^ T, CD8^+^ T and MAIT cells in the same group. p values < 0.05 indicated a significant difference. HD, healthy donor.
