# Supplementary figures and images for: Single-Cell Transcriptomic Profiling of MAIT Cells in Patients With COVID-19
Source: Front Immunol. 2021 Jul 30;12:700152. doi: 10.3389/fimmu.2021.700152 (PMC8363247; doi:10.3389/fimmu.2021.700152)

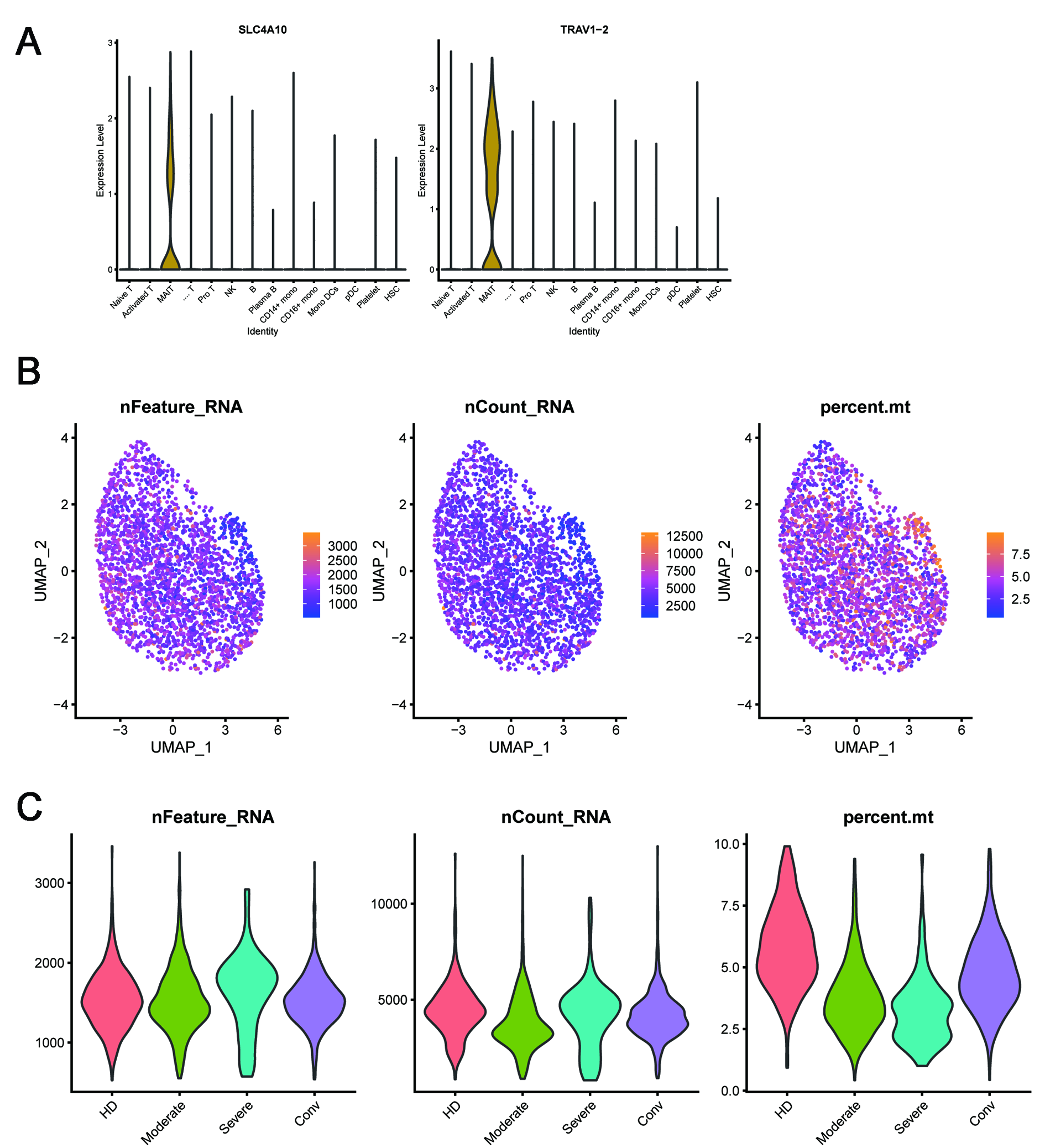

Supplement: Supplementary Figure 1 — Quality of clustering and annotation of MAIT cells. (A) MAIT cell annotation and UMAP projection of integrated single-cell transcriptomes of 121464 cells from all participants. The left two violin plots show the expression distribution of MAIT cell canonical markers in all clusters. The right scatter plot shows all 14 cell types and each dot represents a single cell, colored according to cell type. MAIT cells are circled in red. (B) UMAP projections of gene counts (left), UMI counts (middle), and the percentage of mitochondrial genes (right) in MAIT cells. (C) Violin plots depicting the distribution of gene counts (left), UMI counts (middle), and the percentage of mitochondrial genes (right) in MAIT cells. [file Image_1.tif]

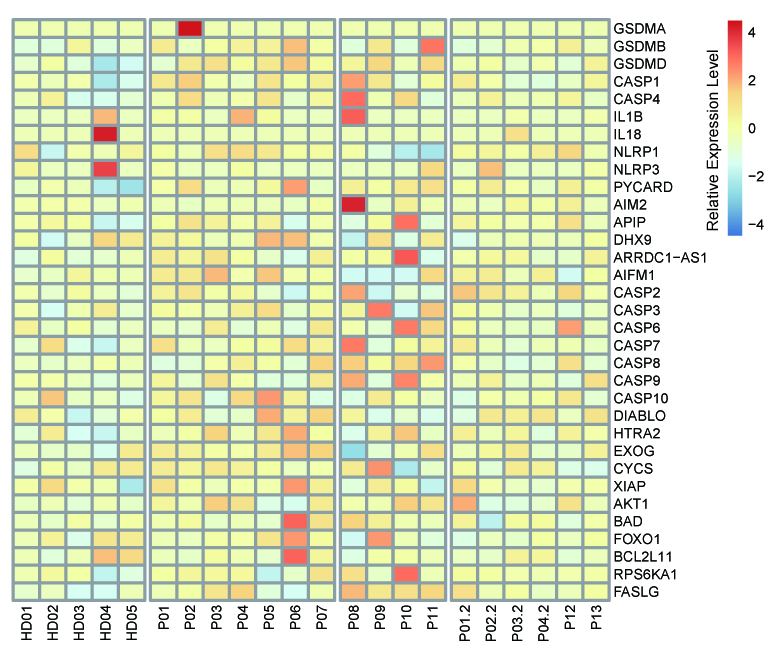

Supplement: Supplementary Figure 2 — Dynamic transcriptional profile of MAIT cells. Heatmap showing the relative expression levels of selected markers in different samples. Rows represent shared genes and columns represent samples from different conditions. [file Image_2.tif]

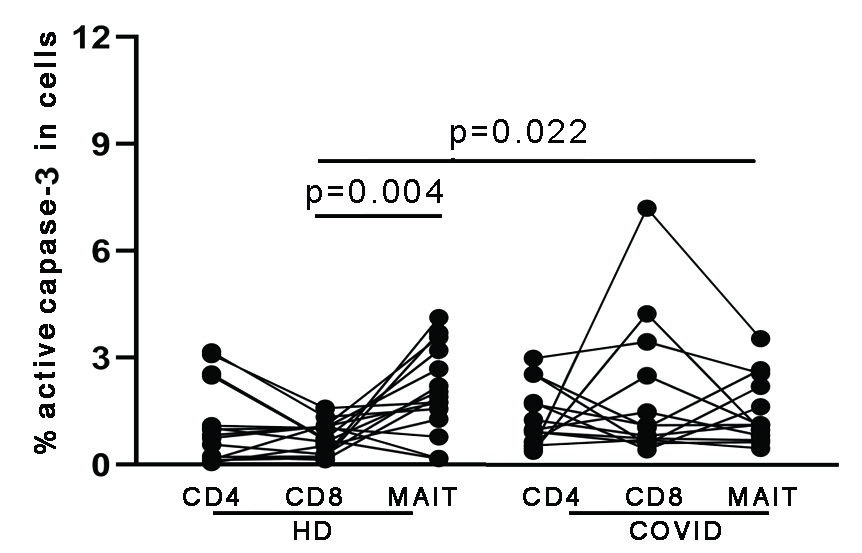

Supplement: Supplementary Figure 3 — Comparisons of active capase-3 in peripheral CD4+ T, CD8+ T and MAIT cells from healthy donors and COVID-19 patients. Statistical analysis was performed using SPSS software version 22 (IBMCorp., Armonk, New York, USA). For comparison, the Mann–Whitney U test was used for comparisons between healthy donors and patients with COVID-19. A paired Student’s t-test was adopted for the analysis of CD4+ T, CD8+ T and MAIT cells in the same group. p values < 0.05 indicated a significant difference. HD, healthy donor. [file Image_3.tif]
