# Supplementary material for: Single-Cell Transcriptomic Profiling of MAIT Cells in Patients With COVID-19
Source: Front Immunol. 2021 Jul 30;12:700152. doi: 10.3389/fimmu.2021.700152 (PMC8363247; doi:10.3389/fimmu.2021.700152)
Supplement: Supplementary file 4 [file Table_1.pdf]

TABLES1 The average expression levels of DEGs (18 genes) in patients with COVID-19

Gene	HD	Moderate	Severe	Conv
**RGCC**	1.8057778	0.3583978	0.368074	0.4496276
**LMNA**	1.4306957	0.2045604	0.3706122	0.2507561
**ZFP36**	3.1116211	2.105539	2.5873654	2.8280009
**IFITM1**	2.5996153	3.6680177	3.6502303	2.7939906
**IFI44L**	0.1219209	1.9507918	1.7254891	0.5390879
**IFI6**	0.4808825	2.0833148	2.0247155	0.7983474
**ISG15**	0.5635043	1.9598343	1.8889938	0.8331265
**XAF1**	0.3536548	1.5144287	1.5013227	0.6306834
**LY6E**	1.3300551	2.2038626	2.1525841	1.4674954
**MX1**	0.2395943	1.216716	1.2287709	0.4884723
**IRF7**	0.2379167	1.0572997	1.2055328	0.4538004
**OAS1**	0.0555059	0.7738829	0.8936585	0.2354653
**EIF2AK2**	0.4184069	1.3384711	1.1712159	0.5026022
**TRIM22**	0.6230162	1.3511981	1.2381396	0.8600541
**TXNIP**	2.1251668	2.7736953	3.1983573	2.5645407
**MT-ND6**	2.0315623	1.2586603	0.9822262	1.5298497
**JUN**	3.1908369	3.523035	2.9550407	3.9216512
**FOS**	3.6319925	2.9654253	2.7075689	3.8156373

For each DEG, they were calculated by averaging the expression levels of cells in HD, Moderate, Severe and Conv condition, respectively. The “AverageExpression” function from Seurat (v.3.2.2) package was used for the calculation of these average expression levels.
